# Supplementary material for: Theophylline Attenuates BLM-Induced Pulmonary Fibrosis by Inhibiting Th17 Differentiation
Source: Int J Mol Sci. 2023 Jan 5;24(2):1019. doi: 10.3390/ijms24021019 (PMC9860752; doi:10.3390/ijms24021019)
Supplement: Supplementary file 1 [file ijms-24-01019-s001.zip › Supplementary Materials_Figure.pdf]

International Journal of Molecular Sciences

MDPI

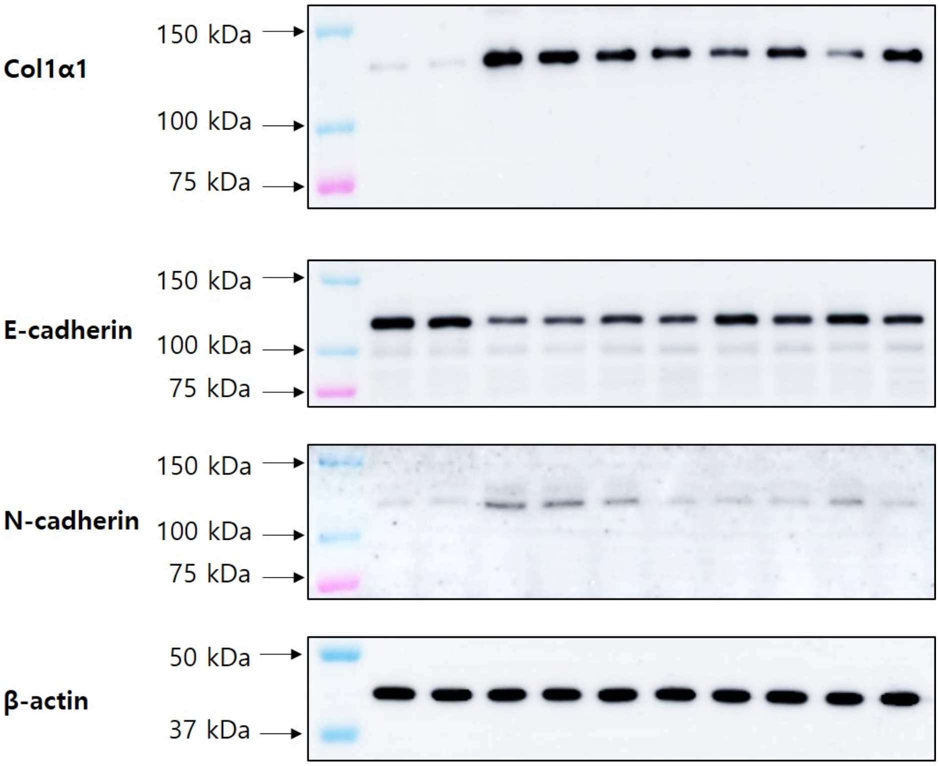

Figure S1. Raw data from western blot in Figure 3D. Uncropped western blot images with marker for protein size were used as data for Figure 3D. Target proteins were noted on the left side of each membrane images.

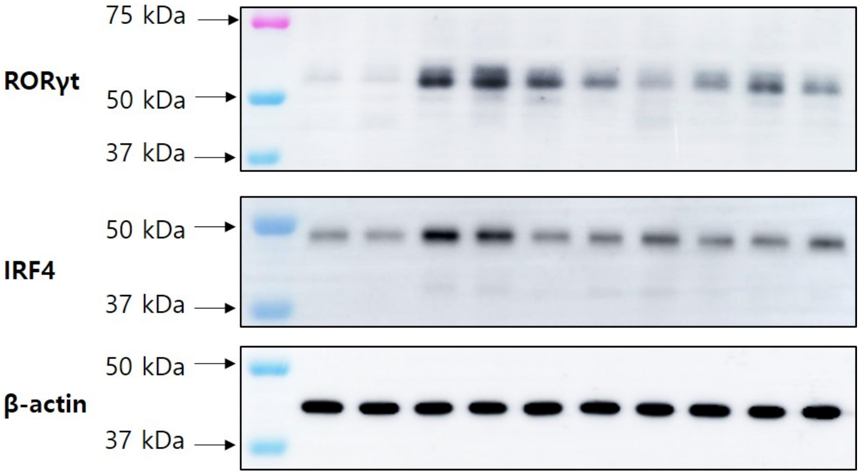

Figure S2. Raw data from western blot in Figure 4E. Uncropped western blot images with marker for protein size were used as data for Figure 4E. Target proteins were noted on the left side of each membrane images.

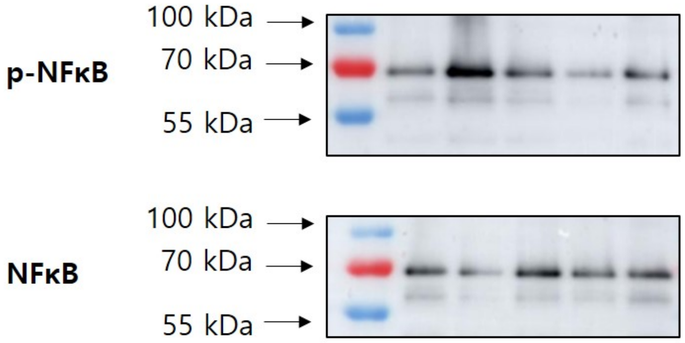

Figure S3. Raw data from western blot in Figure 5B. Uncropped western blot images with marker for protein size were used as data for Figure 4B. Target proteins were noted on the left side of each membrane images.

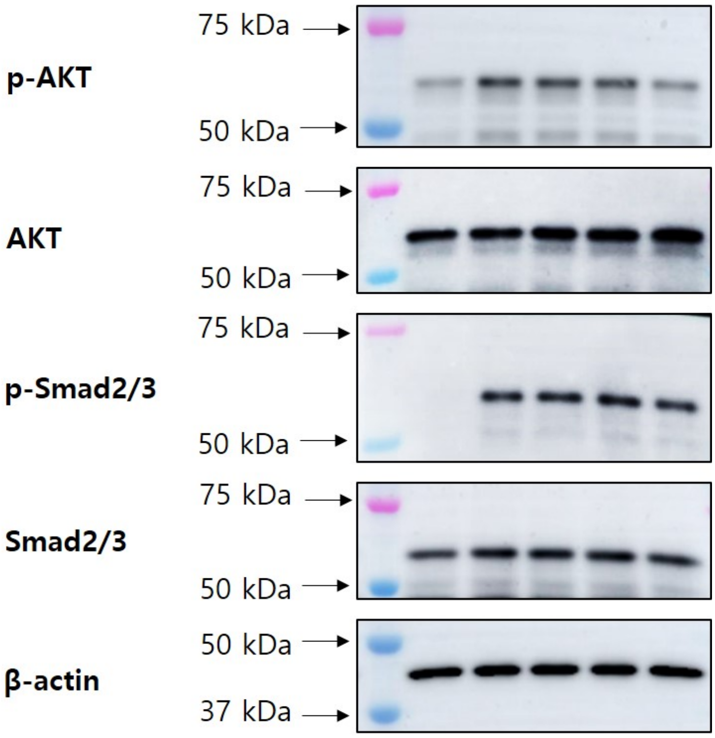

Figure S4. Raw data from western blot in Figure 6C. Uncropped western blot images with marker for protein size were used as data for Figure 4B. Target proteins were noted on the left side of each membrane images.

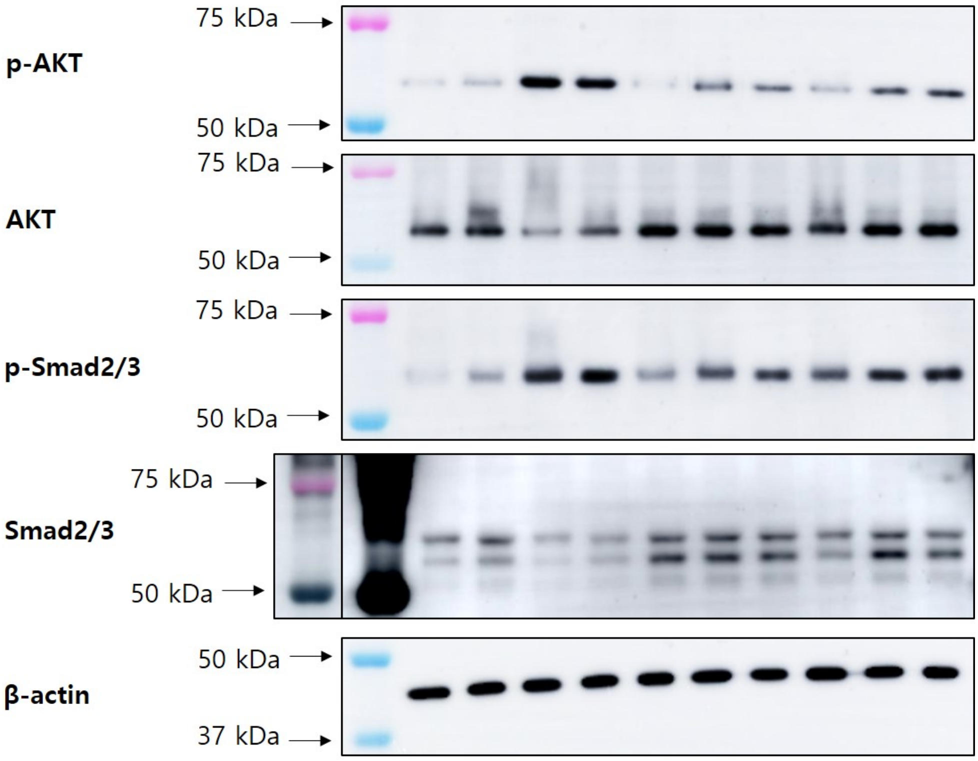

Figure S5. Raw data from western blot in Figure 6D. Uncropped western blot images with marker for protein size were used as data for Figure 4B. Target proteins were noted on the left side of each membrane images.

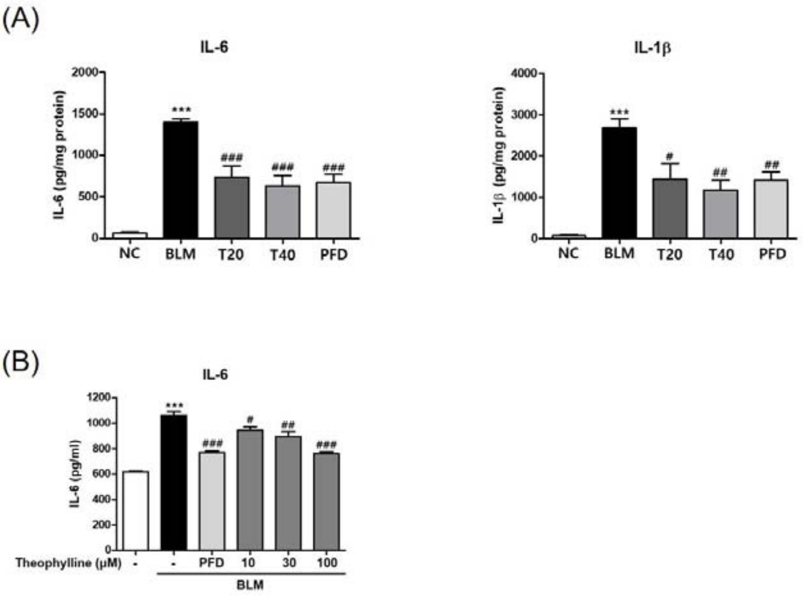

Figure S6. (A) Levels of IL-1β and IL-6 in lung tissues sampled from mice of 5 study groups detected by ELISA related to Figure 4C. (B) Levels of IL-6 in BEAS-2B cells treated with BLM with or without theophylline detected by ELISA related to Figure 5C.
