# Supplementary material for: Theophylline Attenuates BLM-Induced Pulmonary Fibrosis by Inhibiting Th17 Differentiation
Source: Int J Mol Sci. 2023 Jan 5;24(2):1019. doi: 10.3390/ijms24021019 (PMC9860752; doi:10.3390/ijms24021019)
Supplement: Supplementary file 1 [file ijms-24-01019-s001.zip › Supplementary Materials_Table S1.pdf]

Table S1. Sequence of primers and accession number of genes

Genes	Sense (5' to 3')	Antisense (5' to 3')	Size (bp)	Acc #
β-actin (mouse)	GGCTACAGCTTCACCACCACA	CGGATGTCAACGTCACACTT	280	NM_007393
IL-17 (mouse)	CAGCAGCGATCATCCCTCAAAG	CAGGACCAGGATCTCTTGCTG	301	NM_010552
IL-1β (mouse)	TGCCACCTTTTGACAGTGATG	AAGGTCCACGGGAAAGACAC	219	NM_008361
IL-6 (mouse)	TTCCATCCAGTTGCCTTCTT	CAGAATTGCCATTGCACAAC	200	NM_031168
IL-23 (mouse)	AATAATGTGCCCCGTATCCA	CTGGAGGAGTTGGCTGAGTC	213	NM_031252
IRF4 (mouse)	TCTCTGCCAGCCCAGCAGGT	CCTGGGACTCAGGTGGGGCA	211	NM_013674.1
RORgt (mouse)	CGCACCAACCTCTTTTCACG	CAGCTCCACACCACCGTATT	276	NM_001293734.1
TGFβ (mouse)	AGCTGCGCTTGCAGAGATTA	AGCCCTGTATTCCGTCTCCT	189	NM_011577.2
GAPDH (human)	CTCTGCTCCTCCTGTTCGAC	TTCCCGTTCTCAGCCTTGAC	270	NM_002046
Col1α1 (human)	AGTGGTTTGGATGGTGCCAA	ACCCTGGGGACCTTCAGAG	294	NM_000088.3
Col3α (human)	TCCCTGAACACCACCCAGTG	GTGCTTCGTCACCCACGTA	236	NM_001613.2
IL-1β (human)	AGCTACGAATCTCCGACCAC	CGTTATCCCATGTGTCGAAGAA	186	NM_000576
IL-6 (human)	AACCTGAACCTTCCAAAGATGG	TCTGGCTTGTTCCTCACTACT	159	NM_000600
N-cadherin (human)	GAACTGCAAAGCACCTGTGAG	GTGTAGCTCTCGGCGTCAAA	203	NM_004360.4
